# Repurposing cephalosporin antibiotics as pro-senescent radiosensitizers

**DOI:** 10.18632/oncotarget.8984

**Published:** 2016-04-25

**Authors:** Edwardine Labay, Helena J. Mauceri, Elena V. Efimova, Amy C. Flor, Harold G. Sutton, Stephen J. Kron, Ralph R. Weichselbaum

**Affiliations:** ^1^ Department of Radiation and Cellular Oncology, The University of Chicago, Chicago, IL, USA; ^2^ Department of Molecular Genetics and Cell Biology, The University of Chicago, Chicago, IL, USA; ^3^ Ludwig Center for Metastasis Research, The University of Chicago, Chicago, IL, USA

**Keywords:** radiosensitizer, cephalosporin, drug repurposing, senescence, reactive oxygen species

## Abstract

Radiation therapy remains a significant therapeutic modality in the treatment of cancer. An attractive strategy would be to enhance the benefits of ionizing radiation (IR)with radiosensitizers. A high-content drug repurposing screen of approved and investigational agents, natural products and other small molecules has identified multiple candidates that blocked repair of IR damage *in vitro*. Here, we validated a subset of these hits *in vitro* and then examined effects on tumor growth after IR in a murine tumor model. Based on robust radiosensitization *in vivo* and other favorable properties of cephalexin, we conducted additional studies with other beta-lactam antibiotics. When combined with IR, each cephalosporin tested increased DNA damage and slowed tumor growth without affecting normal tissue toxicity. Our data implicate reactive oxygen species in the mechanism by which cephalosporins augment the effects of IR. This work provides a rationale for using commonly prescribed beta-lactam antibiotics as non-toxic radiosensitizers to enhance the therapeutic ratio of radiotherapy.

## INTRODUCTION

Cancer patients with locally advanced tumors who receive radiation therapy frequently receive concurrent chemotherapy to enhance local and distant tumor control [1]. Commonly used cytotoxic drugs including cisplatin, 5-fluorouracil, hydroxyurea, and etoposide are proposed to serve as radiosensitizers, leading to increased local tumor control and improved overall survival in subsets of patients with cervical, anal, head and neck, lung, rectal and breast cancers [2]. However, chemoradiation is commonly associated with significant toxic side effects that are not only dose-limiting but may lead to excess morbidity and mortality. While chemotherapy has the potential to control tumor growth outside the radiation field, the reduced doses employed during chemoradiation may compromise these benefits. Emerging data have established a role for radiotherapy in stimulating anti-tumor immunity to promote both local and systemic control [3-5] but chemoradiation may work at cross purposes to enhanced tumor immunity. Towards identifying alternative agents, a surprisingly diverse range of drugs, nutrients and natural products have been reported to modulate the radiation response of normal tissue and/or tumor cells [6, 7]. While there have been no FDA approvals of non-toxic radiosensitizers to date, among investigational drugs, the poly-ADP-ribose polymerase (PARP) inhibitors are particularly promising candidates [8]. One agent, veliparib, is well-tolerated and has demonstrated radiosensitization in preclinical models [9-11], leading to evaluation in clinical trials [12].

Based on the failure of irradiated cells to resolve ionizing radiation-induced foci (IRIF) when treated with veliparib (ABT-888) [10], we pursued a high-throughput, high-content screen for novel radiosensitizers that would promote IRIF persistence [13]. IRIF are comprised of proteins that assemble within seconds around sites of DNA damage to mediate detection and repair of double strand breaks (DSBs). We tagged IRIF in the human breast cancer cell line MCF7 by expressing green fluorescent protein (GFP) fused to the IRIF binding domain (IBD) of 53BP1 (MCF7^GFP-IBD^) [10] and screened for increased persistence of GFP-IBD foci at 24 hours after IR. We exploited existing collections of approved and investigational drugs, natural products, enzyme inhibitors and other small molecules, hoping to identify non-toxic drugs that might be repurposed as radiosensitizers. The IRIF persistence screen yielded over 100 drugs and other well-studied compounds for further analysis.

Here, toward stratifying drugs identified in the primary screen, we evaluated toxicity and radiosensitization in secondary screens. These analyses reduced the list to 19 drugs which were examined for sensitization of the radioresistant melanoma model B16.SIY [14] yielding cephalexin (Keflex) as a promising hit. Given the significant effectiveness of cephalexin in a preclinical model and the attractive safety profile of the cephalosporin antibiotics, these results support evaluating cephalexin as a clinical radiosensitizer.

## RESULTS

### Stratifying candidate radiosensitizers with an *in vivo* tumor growth delay screen

Our prior repurposing screen [13] identified a wide range of drugs, natural products and neutraceuticals that delayed resolution of IRIF when cells were treated for 1 hour prior to irradiation. We stratified hits based on drug toxicity profiles, ease of administration, chemical diversity, and range of bioactivity, yielding 19 candidates for *in vivo* testing (Table 1). As a tumor model, we used B16.SIY, a radioresistant murine melanoma cell line, injected subcutaneously into the right hind limb of isogenic C57BL/6 mice. Toward identifying hits that display activity at non-toxic doses, the 19 agents were administered to tumor-bearing mice for 2 days before, the day of, and 2 days after a single dose of 15 Gy. As anticipated, tumor growth was delayed compared to IR alone in mice treated with either of the poly(ADP-ribose) polymerase (PARP) inhibitors, rucaparib or veliparib, investigational agents that have been evaluated as radiosensitizers in preclinical models and clinical trials. However, several unanticipated hits similarly slowed tumor regrowth, including cephalexin, CGS15943, clotrimazole, fluoxetine, pitavastatin, resveratrol, synephrine, and trazodone. Based on their broad use in clinical practice and attractive safety profiles, we selected cephalexin (beta-lactam antibiotic), nisoldipine (calcium channel blocker), and trazodone (antidepressant) for further evaluation.

**Table 1:** Radiosensitization of B16.SIY tumors by small molecule inhibitors of IRIF resolution

Compound or generic name	Brand name	Growth delay	Bioactivity	Drug dosing	Source of drug
Cephalexin	Keflex	+++	cephalosporin antibiotic	30 mg/kg twice daily by gavage	MP Biomedicals
CGS15943		+++	adenosine receptor antagonist	3 mg/kg by gavage	Tocris Bioscience
Clotrimazole	Lotrimin	+++	azole antifungal	100 mg/kg by gavage	Alexis Biochemicals
Doxepin	Sinequan	++	tricyclic antidepressant	5 mg/kg, IP	NIH Clinical Collection
Fluoxetine	Prozac	+++	serotonin reuptake inhibitor	10 mg/kg by gavage	Tocris Bioscience
Fluvoxamine	Luvox	+	serotonin reuptake inhibitor	30 mg/kg by gavage	NIH Clinical Collection
Ketotifen	Alaway	++	antihistamine	30 mg/kg, SQ	Enzo Life Sciences
Losartan	Cozaar	++	angiotensin 2 receptor blocker	90 mg/kg by gavage	Santa Cruz Biotechnology
Nisoldipine	Sular	++	calcium channel blocker	60 mg/kg by gavage	Toronto Research Chemicals
Pergolide	Permax	+	dopamine receptor agonist	40 mg/kg by gavage	Tocris Bioscience
Pitavastatin	Livalo	+++	statin	30 mg/kg by gavage	Atamole
Quercetin		++	antioxidant flavonol	30 mg/kg by gavage	Calbiochem
Resveratrol		+++	antioxidant stilbenoid	20 mg/kg by gavage	Santa Cruz Biotechnology
Rucaparib		+++	PARP inhibitor	25 mg/kg twice daily by gavage	AxonMedChem
Synephrine	Oxedrine	+++	adrenergic receptor agonist	3 mg/kg by gavage	LKT Laboratories.
Terbinafine	Lamisil	++	allylamine antifungal	100 mg/kg by gavage	LKT Laboratories
Trazodone	Depyrel	+++	serotonin reuptake inhibitor	25 mg/kg, IP	Toronto Research Chemicals
Trifluoperazine	Stelazine	++	antidopaminergic antipsychotic	0.5 mg/kg, IP	Alexis Biochemicals
Veliparib		+++	PARP inhibitor	25 mg/kg twice daily by gavage	ChemiTek

### Cephalexin, nisoldipine, and trazodone alter DNA damage response *in vitro* and *in vivo*

**Figure 1:**
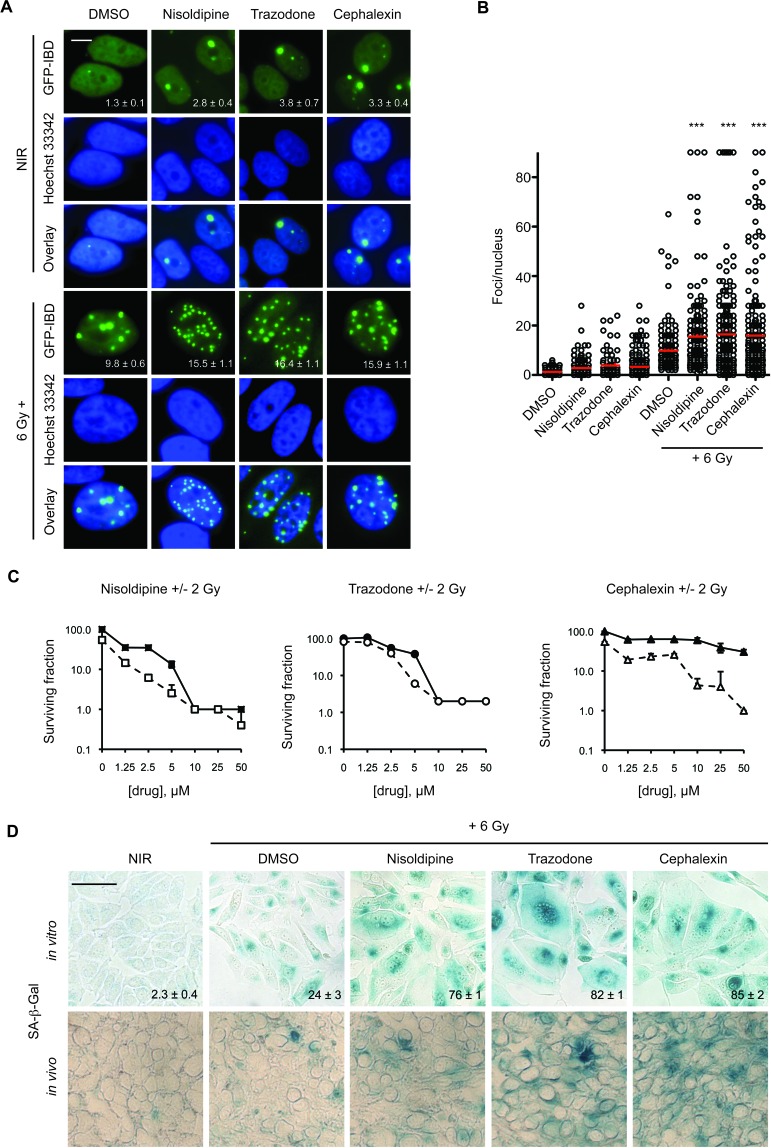
Candidate radiosensitizers induce IRIF persistence, cellular senescence and reduced colony formation in MCF7GFP-IBD cells **A.** Nisoldipine, trazodone and cephalexin block IRIF resolution in MCF7^GFP-IBD^ cells. Cells were treated with 10 μM drug or vehicle for 1 hour prior to IR with 6 Gy. Shown are representative images of non-irradiated cells and 24 hours post IR (GFP-IBD green stain; Hoescht 33342 blue nuclear stain). Foci number per nucleus is reported as mean ± SEM. Scale bar, 10 μm. **B.** Cells were treated as in Figure 1A. Plots of IRIF per nucleus in individual cells are shown, the red bar indicates mean ± SEM. ***, *p* ≤ 0.001 (Mann-Whitney test relative to 6 Gy). **C.** Nisoldipine, trazodone and cephalexin suppress colony formation in MCF7^GFP-IBD^ cells. Representative data from 3 experiments is shown. Solid symbols represent drug alone, open symbols represent drug + 2 Gy. The percent of treatment control ± SEM is reported. **D.** Nisoldipine, trazodone and cephalexin induce cellular senescence in irradiated MCF7^GFP-IBD^ cells and in tumor xenografts. Cells were treated with drug + 6 Gy. Senescence induction was evaluated by SA-β-Gal (blue) staining 5 days post treatment. Percent of SA-β-Gal positive cells are shown and expressed as mean ± SEM (upper panel). Enhanced senescence was also observed in MCF7^GFP-IBD^ tumor tissue sections harvested 5 days post treatment with drug + 6 Gy (lower panel). Scale bar 50 μm.

As initial validation, we reexamined IRIF formation using MCF7^GFP-IBD^ human breast cancer cells. Each drug combined with 6 Gy increased persistence of GFP-IBD foci compared to IR alone at 24 hours (Figure 1A and 1B). Clonogenic assays take into account all modes of death, including but not limited to, apoptosis, necrosis, mitotic catastrophe and senescence. Each drug combined with IR suppressed colony formation at day 9 compared to drug alone (Figure 1C). In apoptosis-resistant MCF7^GFP-IBD^ cells, enhanced cellular senescence was observed following treatment with each drug + 6 Gy compared to IR alone. Cells that display persistent IRIF, suggesting irreparable DNA damage, may withdraw from the cell cycle and develop a senescent phenotype [15]. Senescent cells become enlarged with a flat morphology, permanently lose the ability to proliferate, and exhibit increased senescence associated beta-galactosidase (SA-β-Gal) staining [16]. Senescence induction was verified *in vivo* by treating athymic *nude* mice bearing GFP-MCF7^GFP-IBD^ xenografts with nisoldipine, trazodone or cephalexin plus IR. Enhanced cellular senescence was observed following treatment with drug + 6 Gy compared to 6 Gy alone (Figure 1D).

### Cephalexin, nisoldipine, and trazodone enhance IR sensitivity *in vivo*

**Figure 2:**
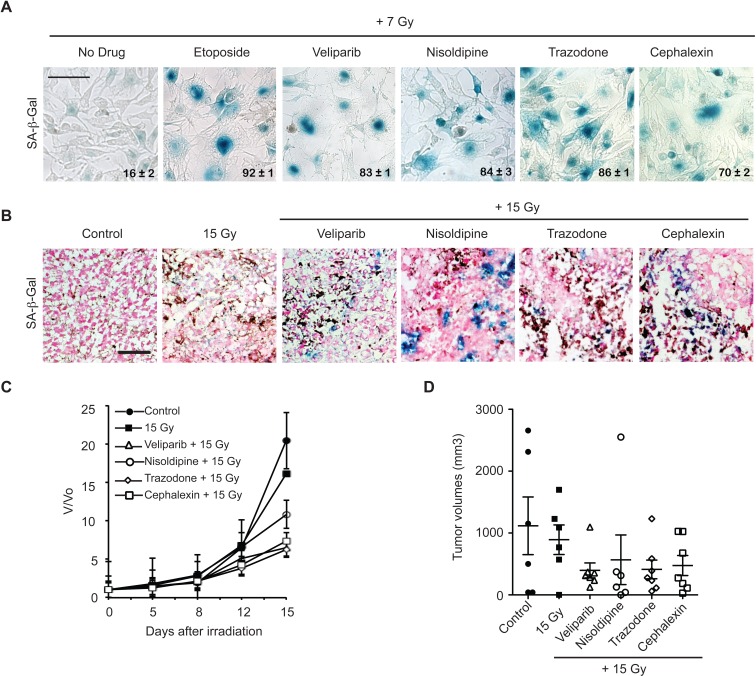
Candidate radiosensitizers induce cellular senescence in B16.SIY murine melanoma cells and tumors and slow tumor growth **A.** Nisoldipine, trazodone and cephalexin induce cellular senescence in irradiated B16.SIY melanoma cells. Cells were treated with 10 μM drug for 1 hour prior to IR with 7 Gy. Senescence induction was evaluated by SA-β-Gal (blue) staining 5 days post IR. Etoposide and veliparib were used as positive controls. Percent SA-β-Gal (+) cells are shown and indicated as mean ± SEM. Scale bar 50 μm. **B.** Nisoldipine, trazodone and cephalexin induce cellular senescence in irradiated B16.SIY tumors. SA-β-Gal activity was determined in tumors sections 7 days after 15 Gy. Veliparib was used as a positive control for senescence. Scale bar 50 μm. **C.** Treatment with nisoldipine, trazodone and cephalexin slowed B16.SIY tumor growth compared to 15 Gy alone. Treatment groups include control (*n* = 7), IR alone (*n* = 6), veliparib + IR (*n* = 8), nisoldipine + IR (*n* = 8), trazodone + IR (*n* = 8), and cephalexin + IR (*n* = 8). **D.** Scatter plot of individual B16.SIY tumors in corresponding treatment groups at day 15 are presented showing the distribution of tumor volumes and the presence of experimental outliers. Mean ± SEM are shown.

To extend the results obtained with MCF7^GFP-IBD^ human breast cancer models, we examined induction of accelerated senescence in B16.SIY murine melanoma cells and tumors. When cephalexin, nisoldipine, or trazodone were combined with 7 Gy, B16.SIY cells displayed accelerated senescence compared to IR alone, much like the positive controls etoposide and veliparib (Figure 2A). Repeating the *in vivo* screen above, C57BL/6 mice bearing B16.SIY tumors were treated with cephalexin, nisoldipine, trazodone, or veliparib 2 days before, the day of and 2 days after a single dose of 15 Gy. Positive SA-β-Gal staining was observed at day 7 post IR in tumors harvested from animals treated with nisoldipine, trazodone or cephalexin (Figure 2B). At day 15, each drug plus 15 Gy delayed tumor growth compared to 15 Gy alone, and to a degree comparable to that observed with veliparib (Figure 2C and 2D).

### Cephalosporin antibiotics delay repair of IR-induced DNA damage and suppress tumor growth

**Figure 3:**
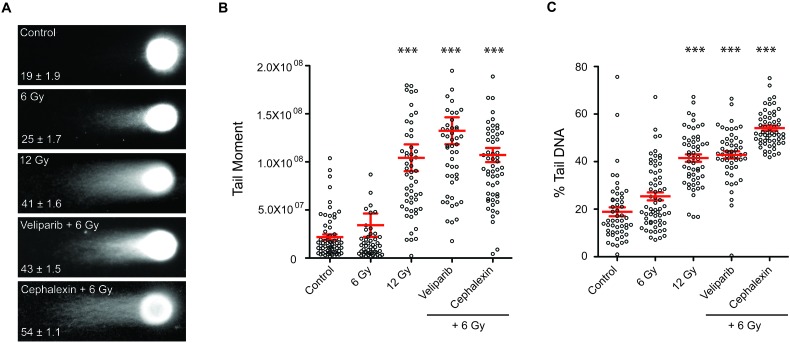
Cephalexin increases persistent DNA damage in irradiated B16.SIY cells **A.** Representative images from neutral comet assay of irradiated B16.SIY cells. Cells were treated with 25 μM veliparib and 50 μM cephalexin. Drugs were administered 1 hour prior to IR. Comet assays were performed 24 hours post IR. **B.** Plots of comet tail moment and **C.** Percent tail DNA with ± SEM are shown. ***, *p* ≤ 0.001, Mann-Whitney test relative to 6 Gy.

Based on *in vitro* and *in vivo* results, we selected cephalexin, a first generation of cephalosporin antibiotic, for further characterization. Confirming broad activity, we observed dose-dependent radiosensitizing effects of cephalexin in MDA-MB-435 breast cancer and SCC61 head and neck cancer cell lines by clonogenic assay (Supplementary Figure 1S). To test if cephalexin affects persistence of IR-mediated DNA double strand breaks (DSB), we performed neutral comet assays on B16.SIY cells. A dose-dependent signal was observed at 24 hours, with greater unrepaired DNA damage detected after 12 Gy compared to 6 Gy (*p* ≤ 0.001, Figure 3A, 3B and 3C). Veliparib significantly increased unrepaired damage after 6 Gy (*p* ≤ 0.001). Like veliparib, cephalexin also significantly increased persistent damage after 6 Gy (*p* ≤ 0.001, Figure 3C), to a level comparable to that observed at 12 Gy.

**Figure 4:**
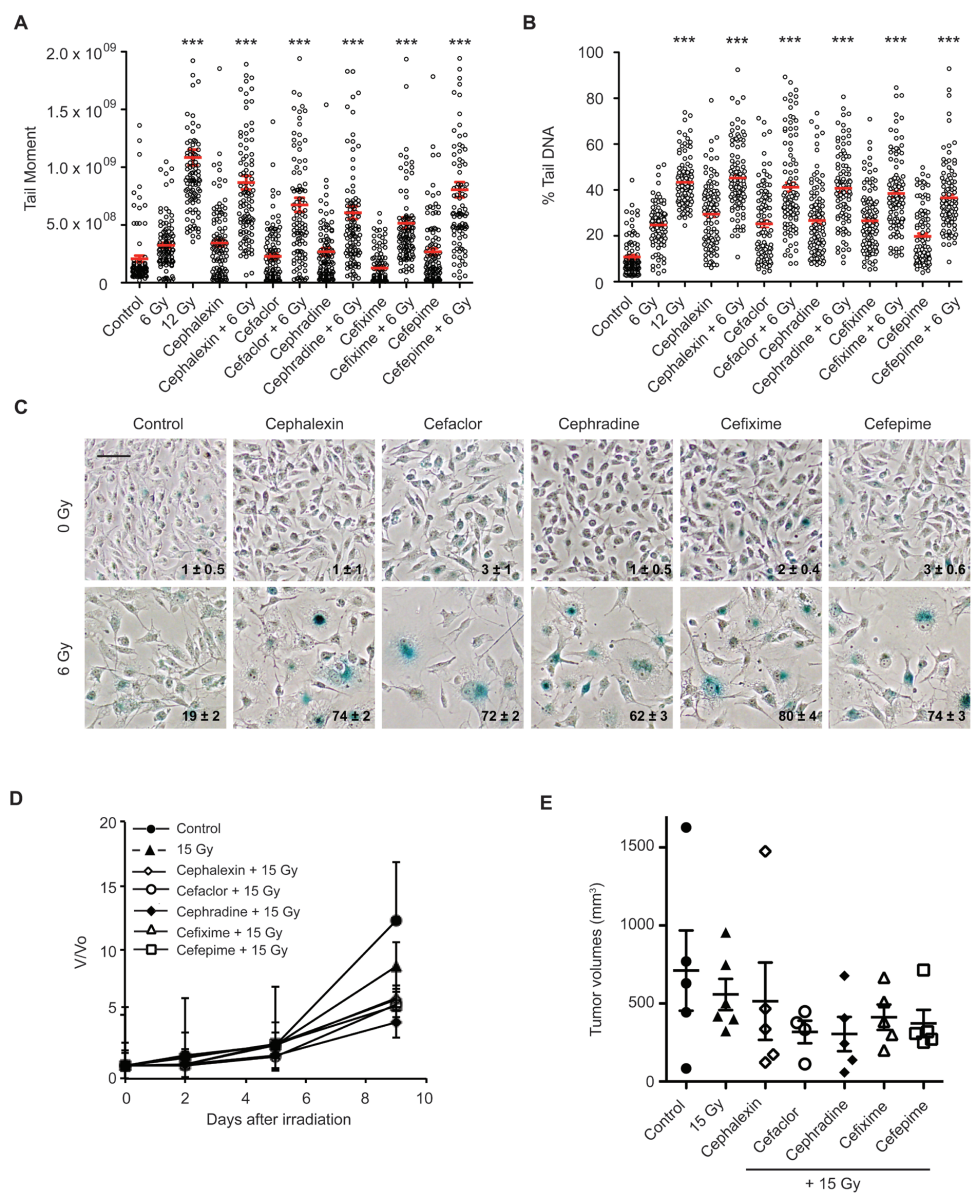
Beta-lactam antibiotics increase DNA damage and senescence in irradiated B16.SIY cells **A.** B16.SIY tumor cells were treated with cephalosporin antibiotics (50 μM) for 1 hour prior to IR with 6 Gy. Comet assays were performed 24 hours later. Plots of comet tail moment and **B.** Percent tail DNA with mean ± SEM are shown. ***, *p* ≤ 0.001, Mann-Whitney test relative to 6 Gy. **C.** B16.SIY tumor cells were treated with drug + 6 Gy and senescence induction was evaluated by SA-β-Gal (blue) staining 5 days later. Cephalosporin antibiotics + 6 Gy produced an increase in cellular senescence compared to drug alone or 6 Gy alone. The percent of SA-β-Gal (+) cells are shown and indicated as mean ± SEM. Scale bar 50 μm. **D.** Cephalosporin antibiotics slowed tumor growth compared to 15 Gy. Groups were Control (*n* = 5), IR alone (*n* = 6), cephalexin + IR (*n* = 5), cefaclor + IR (*n* = 4), cephradine + IR (*n* = 5), cefixime + IR (*n* = 5), and cefepime + IR (*n* = 5). **E.** Scatter plot of individual tumors in corresponding treatment groups at day 9 are presented showing the distribution of tumor volumes and the presence of experimental outliers. Means ± SEM are shown.

Toward mechanism, we examined other cephalosporins for radiosensitization. When cefaclor, cephradine, cefixime, or cefepime were combined with 6 Gy, each drug similarly increased persistent DNA damage by comet assay (*p* ≤ 0.001, Figure 4A and 4B) and produced greater senescence than 6 Gy alone (Figure 4C). Like cephalexin, all four antibiotics slowed tumor growth when combined with 15 Gy compared to radiation alone (Figure 4D and 4E). Neither normal tissue nor systemic toxicity was detected in mice treated with cephalosporin antibiotics alone or in combination with IR.

### Cephalosporin antibiotics increase tumor cell reactive oxygen species

**Figure 5:**
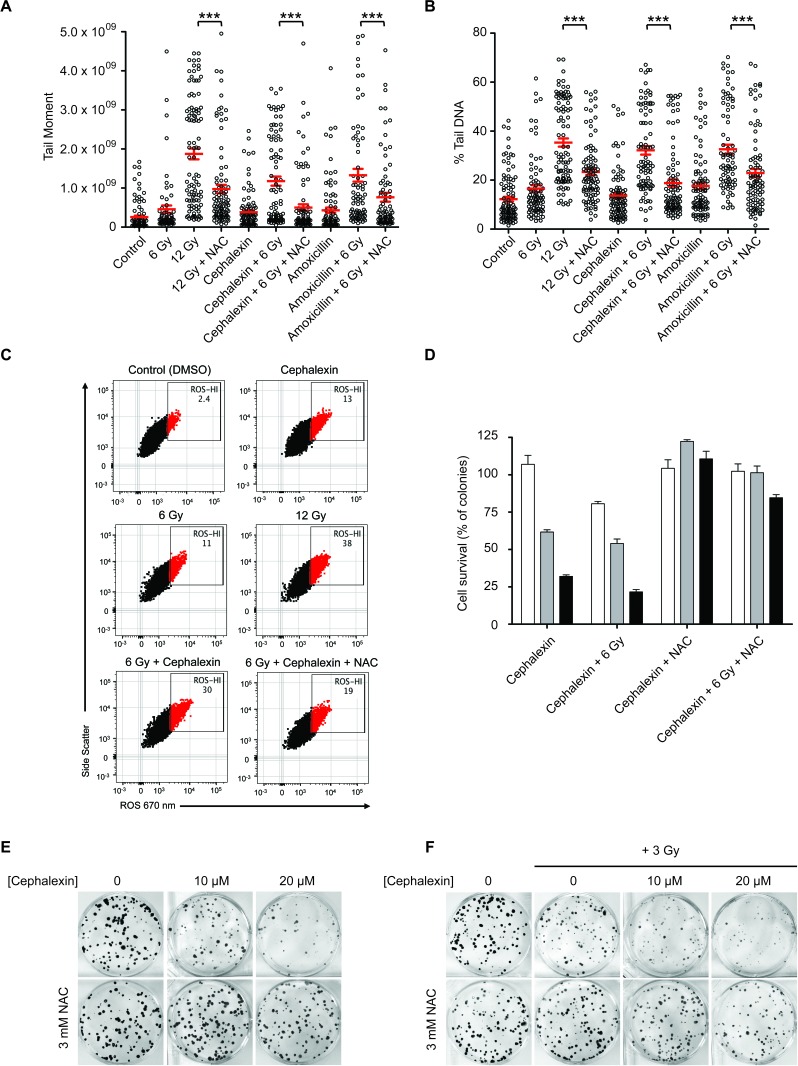
Cephalexin induces ROS production which contributes to radiosensitization **A.** The antioxidant NAC suppresses the effect of beta-lactam antibiotics on DNA damage in irradiated B16.SIY cells. Cells were treated with 50 μM cephalexin or amoxicillin with or without 3 mM NAC 1 hour prior to IR. Comet assay analysis was performed after 24 hours. Plots of tail moment and **B.** Percent tail DNA are presented, with mean ± SEM indicated. ***, *p* ≤ 0.001, Mann-Whitney test. **C.** Cephalexin contributes to increased ROS levels. B16.SIY cells were treated with cephalexin (50 μM), 6 Gy or 12 Gy. Cells were stained with CellROX probe to detect hydroxyl and superoxide radicals, and analyzed by flow cytometry 24 hours later. Approximately 10,000 viable cells are shown per plot. Percent *ROS-HI* cells were gated as shown. ROS induced by cephalexin + 6 Gy was greater than that induced by cephalexin or 6 Gy alone and was reduced by NAC. **D.** NAC suppresses the effect of increasing doses of cephalexin on colony formation of B16.SIY cells. Cells were untreated (white) or treated with cephalexin at 10 μM (grey) or 20 μM (black), with or without 3 mM NAC, 1 hour prior to 3 Gy. Cell survival was evaluated 10 days post treatment. Mean ± SEM are shown. **E.** Clonogenic survival of B16.SIY cells following treatment with cephalexin ± NAC and **F.** with cephalexin ± NAC ± IR, 3 Gy. Representative images of clonogenic assay plates are shown.

Cephalosporins and other beta-lactam antibiotics have been reported to induce mitochondrial dysfunction, resulting in oxidative damage [17, 18]. Collins and colleagues [19] showed that this effect can be suppressed with the antioxidant NAC. To test the potential role for ROS, B16.SIY cells were treated with 3 mM NAC 1 hour prior to cephalexin or amoxicillin as a positive control [20, 21] ± 6 Gy and examined by comet assay after 24 hours (Figure 5A and 5B). Amoxicillin displayed a similar radiosensitizing effect to cephalexin. NAC decreased persistent DNA damage after treatment with amoxicillin + 12 Gy and cephalexin + 12 Gy (*p* ≤ 0.001).

A potential role for ROS in mediating cephalexin anti-tumor effects was confirmed by flow cytometry. B16.SIY cells were treated with 6 Gy or 12 Gy, cephalexin, or cephalexin + 6 Gy. Using a fluorescent reporter, we observed that 6 Gy, 12 Gy or cephalexin alone increased the percentage of cells with high ROS (*ROS-HI*) compared to control. Combining cephalexin and 6 Gy increased *ROS-HI* cells compared to cephalexin alone or IR alone. Pretreatment for 2 hours with 5mM NAC significantly reduced the percentage of *ROS-HI* cells (Figure 5C, *p* ≤ 0.0001). Finally, we performed clonogenic assays on B16.SIY cells. Cells were treated with cephalexin (10 or 20 μM) ± NAC (3 mM) as single agents or in combination with 3 or 6 Gy (Figure 5D, 5E and 5F). Consistent with other results, NAC reduced the cytotoxic effects of both cephalexin and IR.

### Continuous treatment with cephalexin slows B16.SIY tumor growth with or without IR

**Figure 6:**
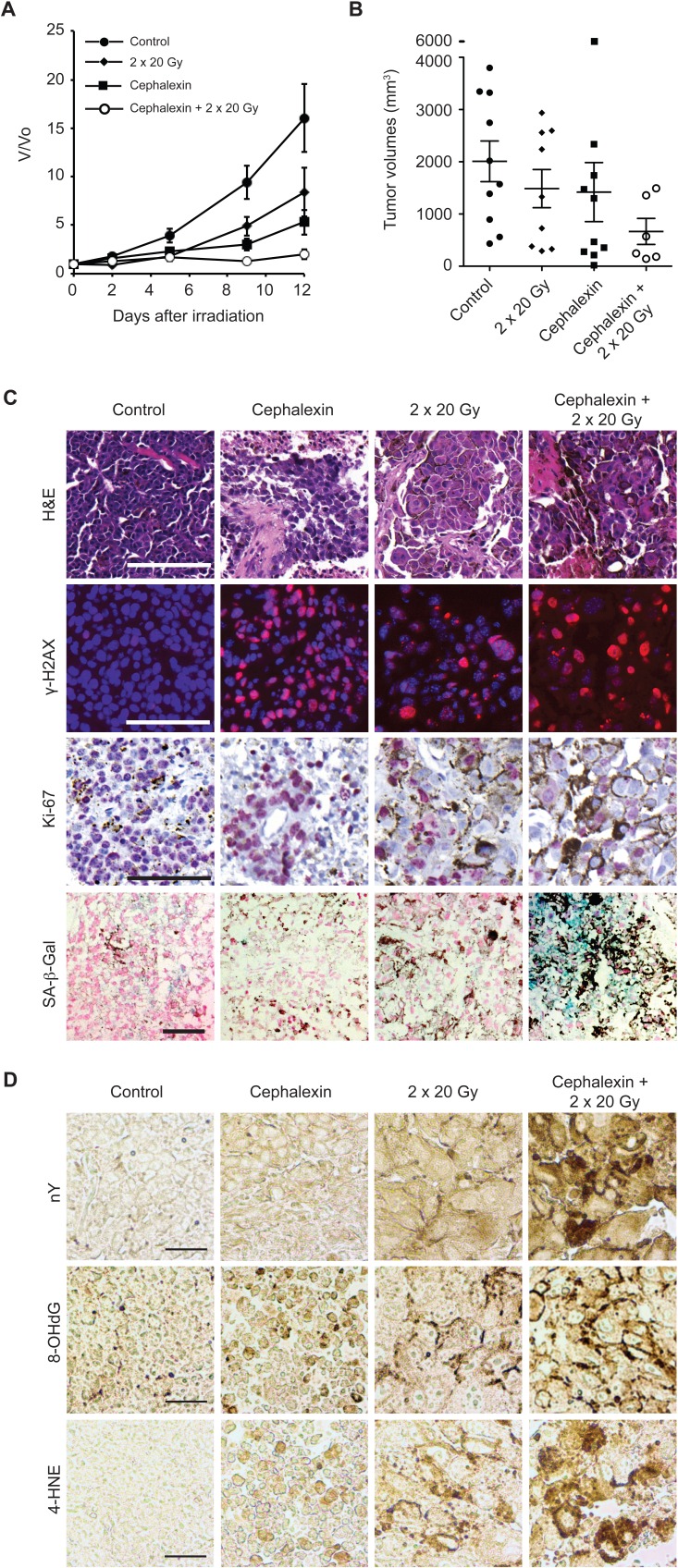
Cephalexin combined with radiation suppresses tumor growth and induces oxidative stress in B16.SIY tumors **A.** Tumor bearing mice were treated for 12 days with cephalexin (30 mg/kg twice daily by gavage) alone or in combination with two doses of 20 Gy. Groups were control (*n* = 10), 20 + 20 Gy (*n* = 10), cephalexin (*n* = 10), and cephalexin + 20 Gy + 20 Gy (*n* = 6). Combined treatment suppressed tumor growth compared to either treatment alone. **B.** Scatter plot of individual tumors in corresponding treatment groups at day 12 showing the distribution of tumor volumes and the presence of experimental outliers. Means ± SEM are shown. **C.** Cephalexin combined with IR produced marked tissue destruction and loss of cellularity. Combined treatment increases DNA damage, suppresses proliferation and induces senescence in B16.SIY tumors. H&E staining revealed extensive tissue destruction 7 days post combined treatment. Treatment with cephalexin + IR further increased the number of γ-H2AX positive cells, decreased the number of Ki-67 positive cells and enhanced SA-β-Gal staining compared to either treatment alone. Representative images are shown. Scale bar 100 μm. **D.** Cephalexin plus IR induce oxidative damage *in vivo.* Tissue sections were examined by immunohistochemistry for oxidative damage to proteins (protein damage marker nitrotyrosine, nY), DNA (DNA damage marker 8-hydroxy-2′-deoxyguanosine, 8-OHdG), or lipids (lipid damage marker 4-hydroxy-2-nonenal, 4-HNE). Compared to control, both cephalexin and radiation increased staining for each oxidative stress marker while the combination produced an increase in each marker suggesting interactive effects. Representative images are shown. Scale bar 100 μm.

To evaluate the potential to translate radiosensitization by cephalexin to the clinic, we examined the effect of multiple IR fractions and prolonged cephalexin treatment. Mice bearing B16.SIY tumors were treated with 30 mg/kg cephalexin twice daily by gavage and/or two 20 Gy fractions 3 days apart. While treatment with cephalexin or radiation alone reduced tumor volume compared to control at 12 days, combined treatment with cephalexin and IR exhibited a combinatorial effect (Figure 6A and 6B).

Histology of treated tumors revealed marked tissue destruction and loss of cellularity after cephalexin alone or IR alone with more pronounced destruction following combined treatment (Figure 6C). Sections were also probed for the DNA-damage marker γ-H2AX, the proliferation marker Ki-67 and senescence marker SA-β-Gal. Treatment with cephalexin or IR increased the number of γ-H2AX positive cells and decreased the number of Ki-67 positive cells compared to control. Combined treatment further increased γ-H2AX positive cells, decreased Ki-67 positive cells and enhanced SA-β-Gal staining compared to either treatment alone.

To survey oxidative damage, tissue sections were also examined by immunohistochemistry for nitrotyrosine (nY), a marker of oxidative protein damage by peroxynitrite, 8-hydroxy-2′-deoxyguanosine (8-OHdG), a marker of oxidative DNA damage due to hydroxyl radicals, and 4-hydroxy-2-nonenal (4-HNE), a marker of lipid peroxidation (Figure 6D). Cephalexin and IR treatment increased immunoreactivity for each marker compared to control while staining following combined treatment was clearly enhanced, suggesting a combinatorial effect between cephalexin and IR.

### Extended treatment with cephalexin is not required for radiosensitization

**Figure 7:**
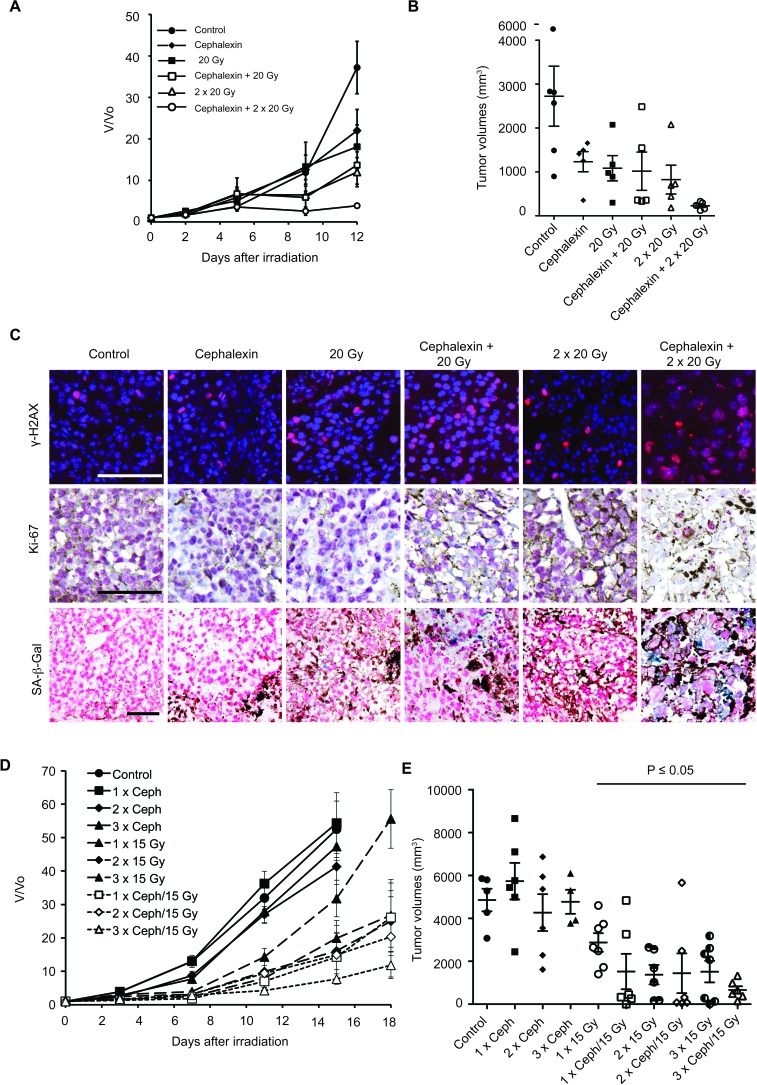
Prolonged treatment with cephalexin is not required to enhance radiosensitizarion and suppress the growth of B16.SIY tumors **A.** Five day treatment of cephalexin (30 mg/kg twice daily) combined with 20 or 40 Gy (20 Gy + 20 Gy) suppressed tumor growth (*n* = 5 per group). **B.** Scatter plot of individual tumors in corresponding treatment groups at day 12 showing the distribution of tumor volumes and the presence of experimental outliers. Mean ± SEM are shown. **C.** Combined treatment with cephalexin + IR increased DNA damage, decreased tumor cell proliferation and enhanced senescence compared to treatment with 20 Gy or 20 Gy + 20 Gy in B16.SIY tumors. Representative images of γ-H2AX, Ki-67 and SA-β-Gal staining are shown. Scale bar 100 μm. **D.** Modeling the integration of cephalexin treatment with hypofractionated therapy indicates feasibility and potential efficacy. One to three cycles of a 3 day course of cephalexin (30 mg/kg twice daily) combined with 15 Gy suppressed tumor growth (10 groups, *n* = 6-7 per group, control, *n* = 5). Mean ± SEM are shown. **E.** Scatter plot of individual tumors in corresponding treatment groups at day 15 showing the distribution of tumor volumes and the presence of experimental outliers. Cycles of cephalexin alone had no significant effect on tumor growth. Significant tumor growth suppression (*p* < 0.05) was observed for IR treatment alone and all combined treatments, except for one outlier in the two cycle group (2 x cephalexin + 15 Gy, *p* = 0.06). Of note for each treatment cycle of cephalexin + IR the number of animals bearing tumor volumes smaller than 1000 mm^3^ was increased compare to all cycles of IR alone.

Toward identifying the minimal dose of cephalexin that induces radiosensitization, mice were treated with 30 mg/kg cephalexin twice daily for 5 days as a single agent or combined with 2 × 20 Gy (interval of 3 days). Combined treatment with cephalexin + IR suppressed tumor growth compared to either treatment alone (Figure 7A and 7B). Combined therapy with cephalexin + IR increased γ-H2AX, decreased Ki-67, and increased SA-β-Gal staining compared to either treatment alone (Figure 7C).

Despite the shorter course of treatment, 5 days of cephalexin was still sufficient to slow tumor growth as a single agent. Toward minimizing direct effects of cephalexin, mice were treated with 30 mg/kg cephalexin twice daily for only 3 days, with or without 15 Gy. After 4 days, animals were treated again, up to a total of three weekly cycles. Tumors treated with cephalexin alone for 1, 2 or 3 cycles and those treated with 15 Gy for 1 or 2 cycles demonstrated a growth pattern similar to untreated controls (Figure 7D). Treatment with 3 × 15 Gy was sufficient to slow tumor growth. Consistent with a combinatorial effect, even a single cycle of combined treatment with cephalexin and 15 Gy produced a significant reduction in tumor growth (*p* ≤ 0.05, unpaired, one-tailed *t*-tests, Figure 7D and 7E). Additional treatment cycles further inhibited tumor growth. We did not detect any local or systemic toxicity in treated animals. Although all tumors eventually regrew, individual mice survived for more than 30 days before requiring euthanasia due to tumor burden.

## DISCUSSION

The development of new cancer therapeutics has become remarkably inefficient, costly, complex and time consuming. Drug repurposing, where new indications are identified for existing drugs, has gained attention in recent years as a practical means to bypass the slow and costly process of introducing new drugs [22-26]. The rationale for repurposing need not be based on implicating the known drug target or a related activity in a new disease. Many drugs exhibit “off target” activities that may be leveraged for beneficial effects [27]. Because drug formulation and safety are already established and efficacy for the new indication may already be documented, a repurposed drug can be moved rapidly into clinical trials or even directly into practice.

Our interest in pursuing a repurposing strategy reflects recent trends in cancer medicine and radiotherapy. In particular, the broader use of hypofractionated ablative radiotherapy modalities such as SABR or SBRT places new constraints on radiosensitizers. Here, patients visit the hospital at weekly intervals to receive image-guided doses of 5 to 20 Gy. Though highly effective and generally well tolerated, local recurrence after hypofractionated therapy is particularly challenging as risks to normal tissue may prevent further irradiation. Thus, radiosensitizers that are highly effective, orally available and offer a high safety margin would be particularly valuable. Suggesting feasibility, multiple prior studies have noted radiosensitization by neutraceuticals or natural products that display little or no genotoxicity on their own [6, 7]. A recent high throughput screen for inhibitors of DSB repair [28, 29] reported identifying a wide range of candidate radiosensitizers including hits from repurposing libraries that lack prior use as cancer therapeutics.

Here, we have built on our prior work that identified over 100 candidate radiosensitizers from repurposing libraries of drugs, natural products, neutraceuticals and other small molecules [13]. These agents were selected by high content screening of tumor cells treated with a single large dose of radiation for ability to block resolution of 53BP1 foci formed at sites of DNA damage and thereby promote onset of accelerated senescence. This focus reflects the increasing recognition that senescence may be a desirable outcome of cancer therapy [30-32]. Though still controversial, the paracrine and/or immunostimulatory effects of senescent cancer and stromal cells have been proposed to contribute to the benefits of genotoxic therapy.

For this study, we stratified the candidate pro-senescent radiosensitizers *via* secondary screens including literature review, cheminformatic analysis, clonogenic survival assays, and comet assays for DNA repair. Thereby, we selected nineteen agents which, along with the positive control veliparib [10], represented a broad range of chemical properties, structures, and reported modes of action. Evaluating these agents in mouse tumor models led us to focus on cephalexin, a cephalosporin beta-lactam antibiotic. As one of the most commonly prescribed generic drugs in the U.S., cephalexin is a particularly promising candidate. Experiments aimed at maximizing therapeutic ratio led us to a treatment schedule of 3 days of 30 mg/kg cephalexin, twice per day by gavage, combined with one 15 Gy dose of radiation on the second day. While neither three days of cephalexin nor a single dose of radiation could slow tumor growth on their own, a single cycle of combined treatment yielded a significant inhibition of tumor outgrowth. In turn, repeating cycles of combined treatment with cephalexin and IR for three weeks slowed tumor growth without toxicity. The evidence for radiosensitization without increased toxicity in a preclinical model argues for evaluating cephalosporins in combination with ablative radiotherapy in the clinic.

We investigated how cephalexin might suppress cancer cell growth and/or enhance the effects of radiation. Collins et al. [19] showed that beta-lactam antibiotics target mitochondria, increasing release of reactive oxygen species (ROS) and resulting in oxidative damage to cellular DNA, protein and lipids. In our work, tumors treated with cephalexin and/or irradiation displayed increased markers of oxidative DNA, protein, and lipid damage, with the greatest effects observed following combined treatment. Collins et al. also observed that pretreatment with NAC, a cell-permeable anti-oxidant, rescued cells from oxidative damage after antibiotics. Similarly, NAC has been shown to block elevated ROS and persistent oxidative stress after radiation. In our study, NAC suppressed the cellular ROS and chromosomal double strand breaks induced by cephalexin, radiation or their combination. These data support a model in which cephalexin increases ROS both as a single agent and when combined with radiation.

A simple model is that increased oxidative damage to chromosomal DNA mediates the apparent synergy between cephalexin and radiation. However, ROS also depletes glutathione and other radioprotective anti-oxidants, and damages nucleotides, other metabolites, RNA, proteins, membranes and organelles. Exposure to ROS also activates oxidative stress signaling pathways, modulates DNA repair, induces unfolded protein response and proteostatic stress, lowers the threshold for apoptosis, and depresses cell survival pathways, all potentially sensitizing cells to subsequent radiation. Multiple such mechanisms may be at work in our experiments.

Taken together, our studies establish cephalosporin antibiotics as promising candidates for repurposing as radiosensitizers. Compared to chemoradiation, cephalosporins offer not only minimal systemic toxicity but may also lower local toxicity at no cost to efficacy. Interestingly, lacking contraindications, cancer patients undergoing ablative radiotherapy may often be prescribed cephalosporins and other beta lactam antibiotics to treat intercurrent infections or as prophylaxis. This raises the possibility that any impacts on the benefits or adverse effects of radiotherapy might be detectable in existing patient data.

## MATERIALS AND METHODS

### Cell lines and cell culture

The MCF7 Tet-On Advanced cell line was obtained from Clontech. The generation and characterization of MCF7^GFP-IBD^ cell line has been previously described [10]. Cells were cultivated less than 20 passages before use. Authenticity was confirmed by short tandem repeat (STR) profile (IDEXX BioResearch) within the last 6 months. Mouse melanoma cell line B16.SIY, a gift of Thomas Gajewski (University of Chicago), was maintained in complete RPMI medium containing 1% penicillin/streptomycin supplemented with 10% FBS.

### Animals and tumor models

Mice were maintained according to guidelines of the Institutional Animal Care and Use Committee and irradiated using a RadSource RS-2000 X-Ray generator operating at 160 kv and 25 mA. Mice were treated with candidate radiosensitizers, described in Table 1 and Table 2, 2 days before, the day of and 2 days after ionizing radiation (IR) unless otherwise indicated. MCF7^GFP-IBD^ tumors established in female athymic *nude* mice (Harlan) as previously [10] were treated once they grew to 300 mm. Female C57BL/6 female mice (Harlan) were injected in the hind limb with 1 × 10^6^ B16.SIY tumor cells suspended in 100 μl PBS. After 8 to 12 days, mice were placed into treatment groups: control, 15 Gy, 2 × 15 Gy, 3 × 15 Gy, 20 Gy, or 2 × 20 Gy, drug alone or drug + IR.

**Table 2:** Radiosensitization of B16.SIY tumors by cephalosporin antibiotics

Compound or generic name	Brand name	Growth delay	Drug doses	Source of drug
Cephalexin	Keflex	+++	30 mg/kg twice daily by gavage	MP Biomedicals
Cefaclor	Ceclor	++	64 mg/kg twice daily by gavage	Alfa Aesar
Cefepime	Maxipime	+	50 mg/kg twice daily by gavage	Alfa Aesar
Cefixime	Suprax	+	10 mg/kg twice daily by gavage	Alfa Aesar
Cephradine	Velosef	++	100 mg/kg twice daily by gavage	R&D Systems

### Clonogenic assays

MCF7^GFP-IBD^ and B16.SIY cells were plated at 100 cells per well in 6 well plates in triplicate in corresponding medium. 24 hours later, drugs were added at a range of concentrations 1 hour prior to IR. Radiation was delivered using a GammaCell ^60^Co source (MDS Nordion). Cells remained in culture for 9-14 days and colonies of at least 50 cells were counted.

### Histology and immunohistochemistry

Formaldehyde-fixed paraffin-embedded (FFPE) tumor sections were stained with hematoxylin and eosin (H&E). Immunohistochemistry for Ki-67 was performed with clone SP6 (Lab Vision), ImmPRESS-AP (Vector Laboratories), Warp Red Chromogen Kit (Biocare Medical) and hematoxylin for counterstaining. Immunofluorescence for γ-H2AX was performed using clone JBW301 (EMD Millipore). Slides were imaged using a Pannoramic slide scanner (Perkin Elmer) equipped with a 40x objective. A representative tumor sample from each group was selected for analysis.

### Detection of DNA damage

For IRIF imaging, MCF7^GFP-IBD^ cells were seeded on cover glass at 2.5 × 10^4^ per well in 24 well plates. GFP-IBD expression was induced with 1 μg/ml doxycycline for 48 hours. Drugs were added for 1 hour prior to irradiation. After 24 hours, cells were fixed, stained with 5 μg/ml Hoechst 33342, mounted using ProLong Gold (Invitrogen) and imaged on an Axiovert 200M microscope with 40X Plan-NeoFluar objective and AxioCam digital camera (Zeiss). Two or more replicates were performed.

For neutral comet assays, B16.SIY cells were seeded at 2 × 10^5^ per well in 6-well plates and treated as above. After 24 hours, cells were mixed with Comet LM agarose and single cell electrophoresis was performed on CometSlides (Trevigen). Slides were fixed, dried, stained with SYBR green and imaged on an Axiovert 40 with a 20X Plan-NeoFluar objective and AxioCam camera. Images were analyzed using an ImageJ comet assay macro (http://www.med.unc.edu/microscopy/resources/imagej-plugins-and-macros/comet-assay). Two or more replicates were performed.

### ROS assays

To examine ROS in tissue culture, B16.SIY cells were seeded in 100 mm culture dishes, incubated overnight and treated with cephalexin (50 μM) for 1 hour prior to 6 or 12 Gy. N-acetyl-cysteine (NAC, 3 mM) was added 1 hour prior to cephalexin as indicated. After 24 hours, cells were washed with PBS and trypsinized. CellROX Deep Red reagent (0.75 μM, Thermo) was added to suspended cells for 60 minutes at 37°C in the dark. Viability stain Sytox Blue (1 μM, Thermo) was added for the last 15 minutes. Stained cells were analyzed with a BD Fortessa flow cytometer and FlowJo software. Dead cells and debris were excluded.

To evaluate ROS in tumors, 5 μm sections from FFPE were blocked in 1% BSA + 5% normal horse serum, probed overnight at 4°C with anti-nitrotyrosine (5 μg/ml, Millipore), anti-8-OHdG (1/200, Abcam) or anti-4-HNE (1/200, Abcam), and detected with secondary-HRP conjugates and DAB (Vector Laboratories). Sections were mounted under PolyMount (Polysciences) and brightfield images were collected using an Axiovert 40 microscope.

### SA-β-Gal assay

Cells were seeded at 3 × 10^4^ per 35 mm Fluorodish (World Precision Instruments). 18 hours later, cells were treated with drug for 1 hour prior to irradiation. Cells were fixed after 5 days and assayed for SA-β-Gal as described [10]. SA-β-Ga positive and negative cells were counted in multiple fields, yielding an average percent SA-β-Gal positive staining, indicated on each SA-β-Gal image as mean ± SEM. Two or more replicates were performed.

To evaluate senescence *in vivo*, 10-12 μm cryosections of OCT-embedded tumors were fixed in 2% paraformaldehyde, stained for SA-β-Gal activity, counterstained with nuclear fast red, dehydrated, mounted and imaged. A representative tumor sample from each group was selected for analysis.

### Statistical analysis

Statistical significance for IRIF counting and comet assays was determined using the non-parametric Mann-Whitney test. Flow cytometric data were analyzed with unpaired, one-tailed *t*-tests. Calculations were performed using Prism (GraphPad) and/or Excel (Microsoft).

## SUPPLEMENTARY MATERIAL FIGURE


