# Supplementary material for: Repurposing cephalosporin antibiotics as pro-senescent radiosensitizers
Source: Oncotarget. 2016 Apr 25;7(23):33919–33. doi: 10.18632/oncotarget.8984 (PMC5085128; doi:10.18632/oncotarget.8984)
Supplement: Supplementary file 1 [file oncotarget-07-33919-s001.pdf]

Repurposing cephalosporin antibiotics as pro-senescent radiosensitizers

Supplementary Material

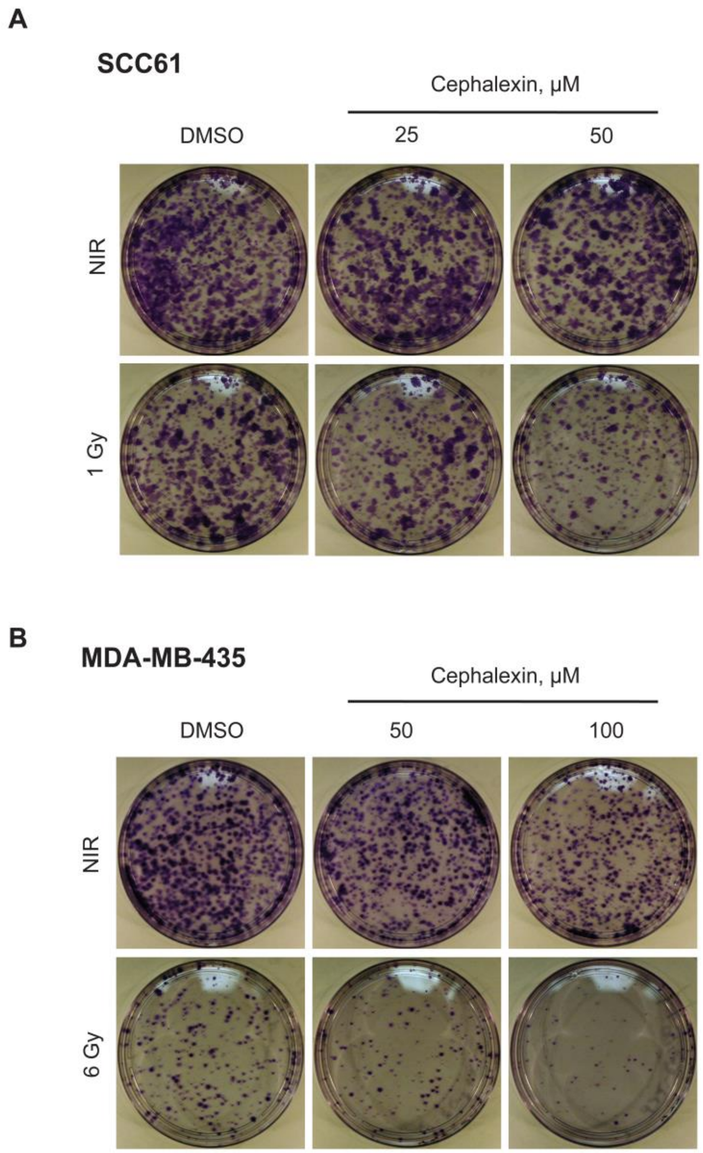

Figure S1: Cephalexin suppresses colony formation in irradiated MDA-MB-435 breast cancer and SCC61 head and cancer cell lines. Cells were untreated or treated with cephalexin 1 hour prior irradiation. Cell survival was evaluated after 14 days. Cells were fixed with ice-cold methanol and stained with crystal violet (0.5% w/v). Representative images of clonogenic assay plates are shown.
